# Programmed Death Ligand 2 Gene Polymorphisms Are Associated With Lung Adenocarcinoma Risk in Female Never-Smokers

**DOI:** 10.3389/fonc.2021.753788

**Published:** 2021-09-24

**Authors:** Sheng-Kai Liang, Li-Hsin Chien, Gee-Chen Chang, Ying-Huang Tsai, Wu-Chou Su, Yuh-Min Chen, Ming-Shyan Huang, Hsien-Chih Lin, Wen-Tsen Fang, Hsiao-Han Hung, Shih-Sheng Jiang, Chih-Yi Chen, Kuan-Yu Chen, I-Shou Chang, Chao A. Hsiung, Chien-Jen Chen, Pan-Chyr Yang, the GELAC Study Group

**Affiliations:** ^1^ Department of Internal Medicine, National Taiwan University Hospital, Hsinchu Branch, Hsinchu, Taiwan; ^2^ Department of Medicine, National Taiwan University Cancer Center, Taipei, Taiwan; ^3^ Division of Biostatistics and Bioinformatics, Institute of Population Health Sciences, National Health Research Institutes, Zhunan, Taiwan; ^4^ Division of Chest Medicine, Department of Internal Medicine, Taichung Veterans General Hospital, Taichung, Taiwan; ^5^ Division of Pulmonary Medicine, Department of Internal Medicine, Chung Shan Medical University Hospital, Taichung, Taiwan; ^6^ School of Medicine, Chung Shan Medical University, Taichung, Taiwan; ^7^ Institute of Medicine, Chung Shan Medical University, Taichung, Taiwan; ^8^ Division of Pulmonary and Critical Care Medicine, Linkou Chang Gung Memorial Hospital, Chang Gung Medical Foundation, Taoyuan, Taiwan; ^9^ Department of Oncology, National Cheng Kung University Hospital, College of Medicine, National Cheng Kung University, Tainan, Taiwan; ^10^ Department of Chest Medicine, Taipei Veterans General Hospital, School of Medicine, National Yang-Ming University, and Taipei Cancer Center, Taipei Medical University, Taipei, Taiwan; ^11^ Department of Internal Medicine, E-Da Cancer Hospital, I-Shou University, Kaohsiung, Taiwan; ^12^ National Institute of Cancer Research, National Health Research Institutes, Zhunan, Taiwan; ^13^ Institute of Medicine, Chung Shan Medical University Hospital, and Division of Thoracic Surgery, Department of Surgery, Chung Shan Medical University Hospital, Taichung, Taiwan; ^14^ Division of Pulmonary and Critical Care Medicine, Department of Internal Medicine, National Taiwan University Hospital and College of Medicine, Taipei, Taiwan; ^15^ Institute of Population Health Sciences, National Health Research Institutes, Zhunan, Taiwan; ^16^ Genomics Research Center, Academia Sinica, Taipei, Taiwan; ^17^ Graduate Institute of Epidemiology, College of Public Health, National Taiwan University, Taipei, Taiwan; ^18^ Institute of Biomedical Sciences, Academia Sinica, Taipei, Taiwan

**Keywords:** programmed death ligand-2, single nucleotide polymorphism, lung adenocarcinoma, pulmonary tuberculosis, carcinogenesis

## Abstract

**Objectives:**

Lung cancer in never-smokers is a distinct disease associated with a different genomic landscape, pathogenesis, risk factors, and immune checkpoint inhibitor responses compared to those observed in smokers. This study aimed to identify novel single nucleotide polymorphisms (SNPs) of programmed death-1 (encoded by *PDCD1*) and its ligands, programmed death ligand 1 (*CD274*) and 2 (*PDCD1LG2*), associated with lung cancer risk in never-smoking women.

**Materials and Methods:**

During September 2002 and July 2012, we enrolled never-smoking female patients with lung adenocarcinoma (LUAD) (n=1153) and healthy women (n=1022) from six tertiary hospitals in Taiwan. SNP data were obtained and analyzed from the genome-wide association study dataset and through an imputation method. The expression quantitative trait loci (eQTL) analysis was performed in both tumor and non-tumor tissues for the correlation between genetic expression and identified SNPs.

**Results:**

A total of 12 *PDCD1LG2* SNPs related to LUAD risk were identified in never-smoking women, including rs2381282, rs4742103, rs4237162, rs4742104, rs12237624, rs78096119, rs6476988, rs7857315, rs10975178, rs7854413, rs56001683, and rs7858319. Among them, six tagged *PDCD1LG2* SNPs rs2381282, rs4742103, rs4237162, rs4742104, rs78096119, and rs56001683 were significantly associated with LUAD risk. Specifically, two *PDCD1LG2* SNPs, rs12237624 and rs78096119, were associated with previous pulmonary tuberculosis infection in relation to LUAD susceptibility. Through an eQTL assay, we found that rs2381282 (*p* < 0.001), rs12237624 (*p* = 0.019), and rs78096119 (*p* = 0.019) were associated with the expression levels of programed death ligand 2.

**Conclusions:**

Novel SNPs of programed death ligand 2 associated with lung adenocarcinoma risk were identified. Among them, two SNPs were associated with pulmonary tuberculosis infection in relation to lung adenocarcinoma susceptibility. These SNPs may help to stratify high-risk populations of never-smokers during lung cancer screening.

## Introduction

Lung cancer is a growing global health concern (1), and cigarette smoking is a well-known risk factor for lung carcinogenesis (2). Nevertheless, approximately 25% of lung cancer cases are not attributable to tobacco smoking, and over 50% of female patients have been reported as never-smokers (3, 4). The prevalence of smoking among women in East Asia is lower compared to that in western countries (5). In Taiwan, more than 90% of female lung cancer patients are never-smokers (6, 7). Lung cancer in never smokers is considered a distinct disease entity with a proteogenomic landscape and oncogenic mechanisms different from those in smokers (8). Therefore, identifying genetic and environmental factors associated with lung cancer risk in never-smokers is urgently needed, especially in Asia.

Inflammation is considered one of the hallmarks of cancer, promoting tumorigenesis and neoplastic progression (9). Chronic infection and inflammation are strongly correlated with cancer risk (10). In addition to cigarette smoking, other environmental factors including chronic inflammation and particle/pollutant inhalation may also play a role in cancer developments (11, 12). However, limited data on the association between chronic infection/inflammation and lung carcinogenesis in never smokers are available.

Inflammation, including immune responses to chronic infection, may help to eliminate abnormal cells and prevent tumorigenesis (13, 14). However, tumors may overcome immune surveillance through mechanisms of immune evasion (9). The programmed death-ligand 1 (PD-L1) or programmed death-ligand 2 (PD-L2) on cancer cells would bind to programmed death-1 (PD-1) on immune cells, which could inhibit T cell activation and proliferation (15–19). PD-1 as a transmembrane protein, which is expressed on activated lymphocytes (T cells, B cells, and tumor specific T cells), natural killer cells, monocytes, and macrophages, involves in the tumorigenesis by restraining immune response (20, 21). The expression of PD-L1 is induced by oncogenes’ expression and various proinflammatory molecules and inhibited by the tumor suppressor genes expression, such as *PTEN* alternations (22). Therefore, activation of PD-1/PD-L1 pathway could lead to immune suppression and promote tumor growth in various cancer types (23, 24).

Further, high PD-L1 expression is not only found to accelerate skin carcinogenesis (25) but also associated with tumor differentiation, vascular invasion, and resistance to epidermal growth factor receptor (EGFR) tyrosine kinase inhibitor treatment in non-small cell lung cancer (NSCLC) (26, 27). In NSCLC, the PD-1/PD-L1 pathway involving tumor proliferation and interacting with tumor microenvironment were greatly investigated. However, the role of PD-L2 in biological function of tumors was rarely studied.

Previous studies reported the association of PD-L1 gene polymorphisms with NSCLC risk mostly in smokers (28, 29). In never-smokers, the roles of PD-1, PD-L1, and PD-L2 gene polymorphisms in lung carcinogenesis remain unclear. Therefore, we conducted a case-control study in never-smoking women to explore the effects of *PDCD1* (encoding PD-1), *CD274* (encoding PD-L1), and *PDCD1LG2* (encoding PD-L2) single nucleotide polymorphisms (SNPs) on lung carcinogenesis. The coding regions of these genes were of particular interest. In this study, chronic obstructive pulmonary disease (COPD), pulmonary tuberculosis (TB) infection, cooking fume exposure, and environmental tobacco smoking were defined as inflammation-related environmental exposures. We specifically investigated the interactions between these environmental factors and SNPs with regard to lung cancer susceptibility.

## Materials and Methods

### Subject Enrollment

This study is a part of the multi-center, case-control Genetic Epidemiological Study of Lung Adenocarcinoma (GELAC) in Taiwan (30–33), which enrolled subjects with lung cancer from six tertiary hospitals between September 2002 and July 2012. Cancer-free individuals were also enrolled as the controls from the health screening centers/clinics of these six hospitals during the recruitment period. Cases diagnosed with primary lung adenocarcinoma (LUAD) confirmed through cytologic or pathologic examination were recruited. Subjects younger than 18 years old, a prior history of other than primary lung cancer, or lack of suitable blood specimen were excluded in this study. We focused on never-smoking female subjects in the GELAC study population. We defined a never smoker as someone who had never smoked or not been smoking at least once a day for more than 6 months at any period during the lifetime. A total of 1153 female LUAD patients and 1022 healthy women were enrolled. The study was approved by the research ethics committees of these six hospitals and the National Health Research Institute in Taiwan.

### Genotyping Analyses

Genomic DNA extracted from blood samples of the study participants was genotyped using an Illumina SNP array (310K, 610K, or 660K). All subjects were included in our previous genome-wide association study (GWAS) (30–33). Furthermore, SNP array and questionnaire data were jointly analyzed for quality control, as done in our previous GWAS. We calculated the relatedness coefficient (PI-HAT) in PLINK (34) and obtained 2175 unrelated samples (PI-HAT < 0.05 for any two samples). SNPs in *PDCD1*, *CD274*, and *PDCD1LG2* were analyzed.

In addition to the retrieved genome-scale genotype data, an imputation was performed by using IMPUTE v.2 and data from the 1000 Genomes Project as the reference panel, so as to obtain more *PDCD1*, *CD274*, and *PDCD1LG2* genotype data (35, 36). After imputation, SNPs derived from the previous GWAS genotype data were filtered in accordance with quality control criteria, including posterior probability > 0.5 and minor allele frequency > 1%. SNPs with a *p*-value < 0.05 in association analysis were selected and annotated as intron, transcript, untranslated region (UTR), missense, or synonymous by using information from the website of University of California, Santa Cruz (https://genome.ucsc.edu/cgi-bin/hgGateway). Micro (mi)RNA-related SNPs were identified using the miRNASNP database (http://bioinfo.life.hust.edu.cn/miRNASNP2/index.php, release 2.0). Tagged SNPs were selected by using Haploview 4.2 (37), a software used to analyze patterns of linkage disequilibrium and haplotypes from genotyping results.

### Clinical Data Collection

All subjects provided written informed consent before collection of blood samples and clinical data. The patients’ clinical characteristics and related information were previously described (30–33). Clinical data were obtained from medical records as well as through personal interviews based on questionnaires and included age, education levels, body mass index (BMI, kg/m2), smoking status (including active and passive smoking), COPD, previous pulmonary TB infection, cumulative duration of hormone replacement therapy and contraceptive medications, and cooking fume exposure.

The body weight of healthy controls was recorded to adjust for the interference of cancer-related weight loss in BMI estimation. The BMI values were categorized into five levels (< 18.5, 18.5-24, 24-27, 27-30, and ≥ 30) and treated as categorical variables, following the guideline of Taiwan’s Administration of Health Promotion. A subject who had been smoking cigarettes regularly for at least 6 months, regardless of whether she had now quit or not, was defined as an “ever-cigarette smoker”. Otherwise, the subject was defined as a never-smoker (38, 39).

Supplementary therapy with synthetic estrogen or/and progesterone for a period of more than 90 days was defined as hormone replacement use. Contraceptive use was defined as the use of relevant medication for over 90 days on a cumulative basis. Cooking fume exposure was defined as a history of continuous cooking for more than 180 days. Furthermore, the cumulative cooking fume exposure (the duration of cooking is defined in years) was calculated by multiplying the number of cooking times every day by the number of cooking years. Cooking without a fume extractor was defined as the subject being continuously exposed to cooking fumes for at least 6 months without using a fume extractor.

Educational degree was considered a variable with 6 levels of value: 1 for lower than elementary school, 2 for elementary school graduate, 3 for junior high school graduate, 4 for senior high school graduate, 5 for college graduate, and 6 for postgraduate education. Exposure to environmental tobacco smoking (ETS) was categorized as being from parents or spouse, other relatives, and workplace, which were also assessed and stratified.

### Quantitative Trait Loci Expression

The identified SNPs associated with LUAD risk were assessed for their association with the mRNA levels of the respective genes by quantitative trait loci (eQTL) assay. This *cis* eQTL analysis was performed using the Lung Cancer Tissue Cohort of Never-smokers, which included 115 never-smoking LUAD patients from the China Medical University Hospital in Taiwan. We collected their tumor tissues, adjacent non-tumor tissues, blood, and clinical information. Microarray gene expression experiments were performed, and genome-scale genotype data based on buffy coat DNA were obtained. Details are available from our previous study (GSE46539) (33).

### Statistical Analysis

Logistic regression models were applied to assess the relationship between each selected covariate and LUAD risk. To investigate the correlation between individual SNPs and LUAD risk, we introduced clinical variables with a *p*-value less than 0.05 into multivariate logistic regression analysis. We coded the genotypes as additive by using the counts of the minor allele for each SNP.

For categorical clinical risk factors with more than two levels (more than one *p*-value in a model, such as BMI), the factor was retained if one of the *p*-values was less than 0.05. Two-tailed tests were used to determine significance in all analyses. A *p*-value less than 0.05 was considered statistically significant for identifying correlations between SNPs and LUAD risk. The interactions were estimated by including additional interaction terms (each SNP × inflammation-related environmental factors) in the logistic models. Statistical tests were performed by using R, a free software of the GNU project.

## Results

### Clinical Characteristics

A significant association with LUAD risk was observed for low education levels (*p* < 0.001), any first-degree family member with a history of lung cancer (*p* < 0.001), previous pulmonary TB infection (*p* < 0.001), cooking time in years (*p* = 0.037), cooking fume exposure (*p* < 0.001), and exposure to ETS from relatives or workplace (*p* < 0.001) (**Table 1**). Covariates, including age, education levels, BMI levels, any first-degree family with a history of lung cancer, previous pulmonary TB infection, cooking time, cooking with a fume extractor, and ETS exposure, were thus introduced into multivariate analyses.

**Table 1:** Clinical characteristics of lung adenocarcinoma patients and healthy controls.

n (%)	Cases	Controls	*p* ^b^
	(n = 1153)	(n = 1022)	
Age (years old), mean (SD)	59.63 (11.34)	58.96 (11.06)	0.162
Education levels			< 0.001
Lower than elementary school	255 (22.14)	157 (15.38)	
Elementary school graduate	383 (33.25)	326 (31.93)	
Junior high school graduate	136 (11.81)	144 (14.10)	
Senior high school graduate	215 (18.66)	201 (19.69)	
College graduate	154 (13.37)	167 (16.36)	
Postgraduate	9 (0.78)	26 (2.55)	
BMI^a^			
< 18.5	47 (4.17)	29 (2.88)	Baseline
18.5–24	597 (52.97)	519 (51.59)	0.159
24–27	307 (27.24)	290 (28.83)	0.088
27–30	112 (9.94)	124 (12.33)	0.030
≥ 30	64 (5.68)	44 (4.37)	0.724
First-degree family with lung cancer			< 0.001
Yes	134 (12.96)	43 (5.93)	
No	900 (87.04)	682 (94.07)	
COPD			0.174
Yes	26 (2.28)	15 (1.48)	
No	1112 (97.72)	1001 (98.52)	
History of pulmonary TB infection			< 0.001
Yes	50 (4.39)	17 (1.67)	
No	1088 (95.61)	1001 (98.33)	
HRT use			0.399
Yes	197 (18.12)	188 (19.58)	
No	890 (81.88)	772 (80.42)	
Contraceptive use			0.630
Yes	77 (7.15)	75 (7.71)	
No	1000 (92.85)	898 (92.29)	
Cooking time in years, mean (SD)	74.29 (53.22)	69.59 (50.47)	0.037
Cooking without fume extractor			< 0.001
Yes	68 (5.9)	23 (2.25)	
No	1085 (94.1)	999 (97.75)	
ETS exposure			< 0.001
Yes	856 (77.05)	668 (66.73)	
No	255 (22.95)	333 (33.27)	

BMI, body mass index; COPD, chronic obstructive pulmonary disease; ETS, environmental tobacco smoking; HRT, hormone replacement therapy; SD, standard deviation; TB, tuberculosis.

a Body weight (kg)/body height (m^2^).

b p values for continuous variables (age, education levels, and cooking time in years) were determined via two-sample t-test; p-values for binary variables were determined via Chi-square test. For BMI, a categorical clinical risk factor with five levels, p-values were determined from the multivariate logistic regression model with BMI < 18.5 as the baseline.

### *PDCD1LG2* SNPs Are Associated With LUAD Risk

**Figure 1:**
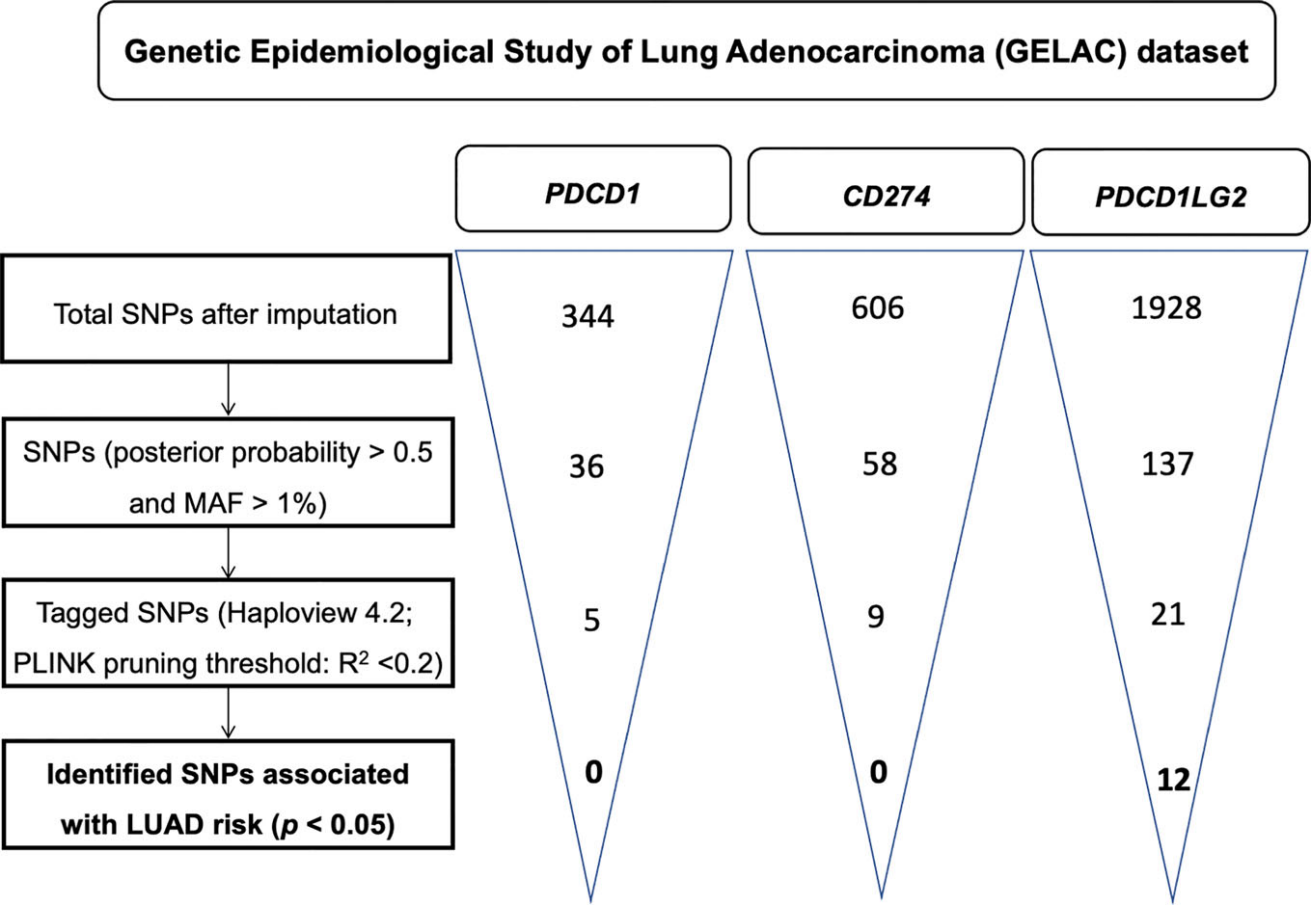
A flowchart for the identification of single nucleotide polymorphisms (SNPs) associated with the risk of lung adenocarcinoma in *PDCD1*, *CD274*, and *PDCD1LG2*.

**Figure 2:**
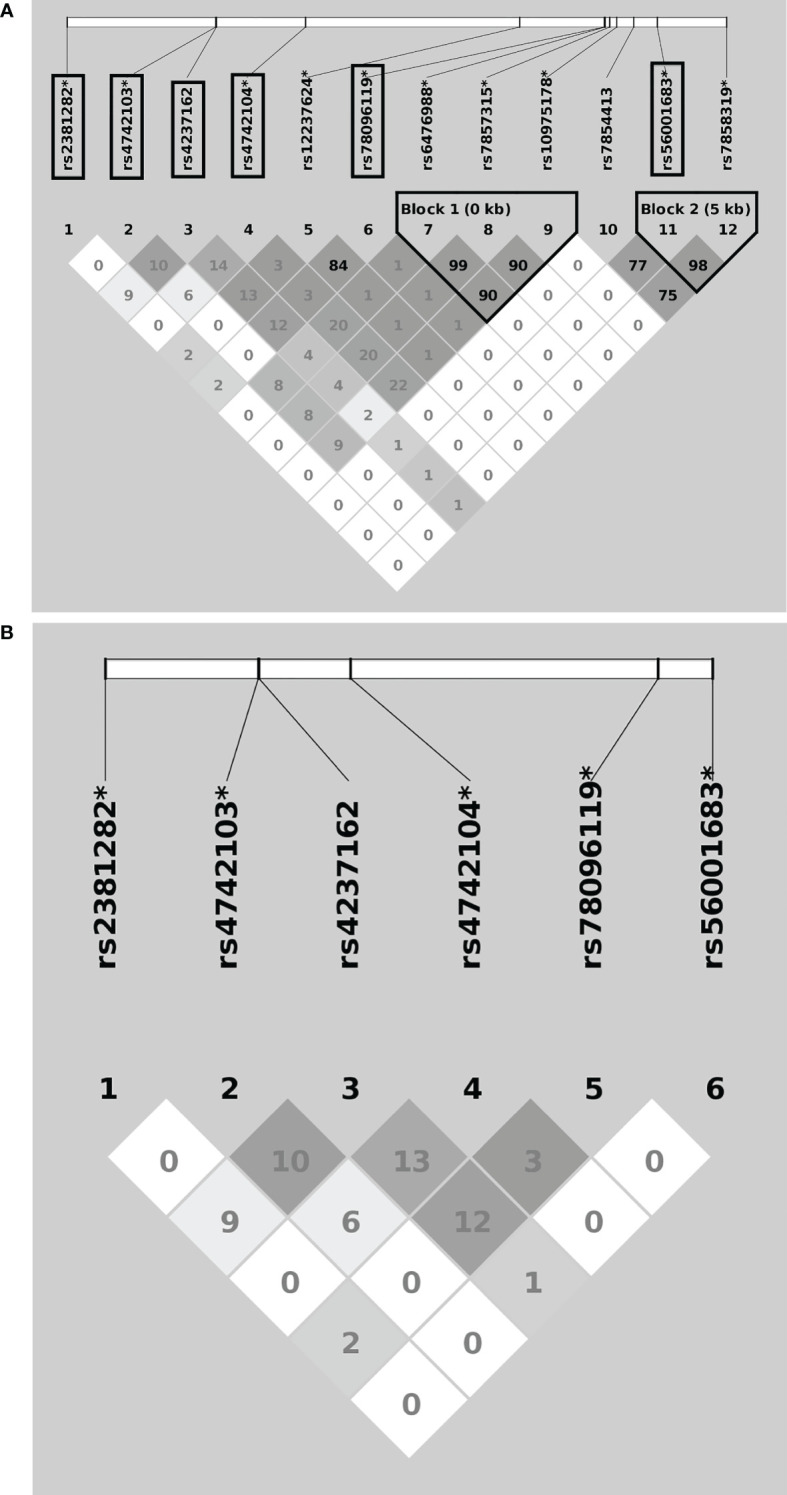
Linkage disequilibrium patterns of the overall 12 **(A)** and the 6 **(B)** tagged *PDCD1LG2* single nucleotide polymorphisms (SNPs) associated with lung adenocarcinoma risk. The “*” indicates imputed SNPs, and the frames indicate the tagged SNPs.

A flowchart for the identification of *PDCD1, CD274*, and *PDCD1LG2 SNPs* associated with LUAD risk is presented in **Figure 1**. There were 36, 58, and 137 genotyped SNPs located within *PDCD1*, *CD274*, and *PDCD1LG2*, respectively, all of which met the genotype control criteria (**Supplementary Table S1**). No *PDCD1* SNPs were associated with LUAD risk. One *CD274* SNP rs144841978 was related to LUAD risk with borderline significance (*p* = 0.051) and annotated in the 3′-UTR as a non-coding transcript variant. A total of 12 *PDCD1LG2* SNPs were significantly associated with the LUAD risk, including rs2381282, rs4742103, rs4237162, rs4742104, rs12237624, rs78096119, rs6476988, rs7857315, rs10975178, rs7854413, rs56001683, and rs7858319 (**Table 2**). Among them, rs2381282, rs4742103, rs4237162, rs4742104, rs78096119, and rs56001683 were further identified as tagged SNPs (**Figure 2A**). The linkage disequilibrium (LD) patterns of these tagged SNPs are shown in **Figure 2B**.

**Table 2:** *PDCD1LG2* SNPs associated with lung adenocarcinoma risk.

SNP	Allele^a^	MAF	β	OR (95% CI)	*p* ^b^	Annotation
rs2381282^#^	T/C	0.357	0.149	1.160 (1.001, 1.345)	0.049	intron_variant
rs4742103^#^	C/T	0.304	-0.221	0.802 (0.676, 0.950)	0.011	intron_variant
rs4237162	C/T	0.221	0.235	1.264 (1.058, 1.511)	0.010	intron_variant
rs4742104^#^	C/T	0.425	-0.185	0.831 (0.715, 0.966)	0.016	intron_variant
rs12237624^#^	C/T	0.043	0.390	1.476 (1.033, 2.111)	0.033	intron_variant
rs78096119^#^	A/G	0.047	0.365	1.440 (1.027, 2.020)	0.035	intron_variant
rs6476988^#^	A/G	0.273	0.166	1.181 (1.003, 1.390)	0.046	intron_variant
rs7857315^#^	T/C	0.273	0.176	1.192 (1.013, 1.404)	0.035	intron_variant
rs10975178^#^	A/G	0.294	0.178	1.195 (1.019, 1.402)	0.028	intron_variant
rs7854413	C/T	0.101	-0.294	0.745 (0.588, 0.945)	0.015	missense_variant I (ATA) –> T (ACA) synonymous_variant D (GAT) –> D (GAC)
rs56001683^#^	G/T	0.109	-0.240	0.786 (0.627, 0.987)	0.038	intron_variant
rs7858319^#^	C/A	0.108	-0.241	0.786 (0.625, 0.988)	0.039	intron_variant

CI, confidence interval; MAF, minor allele frequencies; OR, odds ratio; SNP, single nucleotide polymorphism; UTR, untranslated region.

a Minor/major allele.

b Covariates of age, education levels, body mass index, first-degree family with a history of lung cancer, history of pulmonary tuberculosis infection, cooking time in years, cooking with fume extractor, and environmental tobacco smoking exposure were used as adjusted variables.

^#^Imputed SNP.

### *PDCD1LG2* SNPs rs12237624 and rs78096119 Were Associated With Previous Pulmonary TB Infection in Relation to LUAD Susceptibility

Since the PD1/PD-L1/PD-L2 pathway plays a critical role in the anti-tumor immune response, we further investigated the interaction between identified SNPs and inflammation-related environmental factors, including COPD, history of pulmonary TB infection, cooking time, cooking with a fume extractor, and ETS exposure.

Associations between previous pulmonary TB infection and *PDCD1LG2* SNPs rs12237624 and rs78096119 in relation to LUAD risk were observed (**Supplementary Table S2**). The LD between these two SNPs was 0.84 (R^2^). No subject had two minor alleles for these SNPs and a history of pulmonary TB infection. We treated the presence of two minor alleles as a single category during association analysis (the dominant model). For rs12237624, this risk allele was significantly associated with an increased LUAD risk among patients with a history of pulmonary TB (OR_TT_ = 3.605, 95% CI = 1.688 - 7.699) (*p* < 0.001) (**Table 3**), as was the case for SNP rs78096119 (OR_GG_ = 4.075, 95% CI =1.842 - 9.014) (*p* < 0.001) (**Table 3**). Otherwise, no significant association with the other inflammation-related environmental factors was observed.

**Table 3:** Correlation between *PDCD1LG2* SNPs and pulmonary TB for lung adenocarcinoma susceptibility.

SNP	Genotype/TB	Case	Control	Odds Ratio	Adjusted *p*-value^a^	*p*-value for correlation^b^
rs12237624	TT + no TB	987 (86.88%)	930 (91.45%)	1		0.040
	CT/CC + no TB	99 (8.71%)	70 (6.88%)	1.975 (0.392 - 9.938)	0.409	
	TT + TB	46 (4.05%)	13 (1.28%)	3.605 (1.688 - 7.699)	< 0.001	
	CT/CC + TB	4 (0.35%)	4 (0.39%)	NA	NA	
rs78096119	GG + no TB	978 (86.09%)	925 (90.95%)	1		0.031
	AG/AA + no TB	109 (9.60%)	75 (7.37%)	1.957 (0.392 - 9.772)	0.413	
	GG + TB	46 (4.05%)	11 (1.08%)	4.075 (1.842 - 9.014)	< 0.001	
	AG/AA + TB	4 (0.35%)	5 (0.49%)	NA	NA	

NA, data not available; SNP, single nucleotide polymorphism; TB, tuberculosis.

a Adjusted for education level, cooking time, and passive smoking.

b The p-values were obtained from the additive model (**Supplementary Table S2**). Covariates of age, education levels, body mass index, first-degree family with a history of lung cancer, history of pulmonary tuberculosis infection, cooking time in years, cooking with fume extractor, and environmental tobacco smoking exposure were used as adjusted variables.

### Expression of Quantitative Trait Loci

eQTL analyses were performed for the aforementioned *PDCD1LG2* SNPs (**Supplementary Table S3**). The significance of eQTL results in tumor or non-tumor tissues was determined on the basis of *p* < 0.05 as the threshold. Among the *PDCD1LG2* SNPs, rs2381282 (*p* < 0.001), rs12237624 (*p* = 0.019), and rs78096119 (*p* = 0.019) risk alleles were negatively associated with PD-L2 expression in non-tumor tissues.

## Discussion

In this multi-center case-control study, a total of 12 *PDCD1LG2* SNPs (rs2381282, rs4742103, rs4237162, rs4742104, rs12237624, rs78096119, rs6476988, rs7857315, rs10975178, rs7854413, rs56001683, and rs7858319) associated with LUAD risk in never-smoking women were identified. Among them, rs2381282, rs4742103, rs4237162, rs4742104, rs78096119, and rs56001683 were recognized as tagged SNPs. Furthermore, *PDCD1LG2* SNPs rs12237624 and rs78096119 had significant associations with a history of pulmonary TB infection related to LUAD susceptibility. The *PDCD1LG2* SNPs rs12237624, rs78096119, and rs2381282 were associated with PD-L2 expression *via* eQTL analysis. To our knowledge, this is the first study identifying novel PD-L2 gene polymorphisms associated with lung carcinogenesis in female never-smokers.

Among the 12 *PDCD1LG2* SNPs, the clinical significance of rs7854413 was the most commonly reported in the previous literatures. A cohort study in south India reported that patients with the *PDCD1LG2* SNP rs7854413 and lymphatic filariasis infection were susceptible to chronic lymphatic pathologies (40), and rs7854413 polymorphism was related to advanced fibrosis and development of hepatocellular carcinoma from patient with non-alcoholic steatohepatitis (41). Notably, rs7854413 was also associated with recurrence in patients with early-stage NSCLC (42). During the process of literature review, no studies on these *PDCD1LG2* SNPs other than rs7854413 were reported. The functional role of these SNPs in lung carcinogenesis warrants further investigation.

Immune checkpoint blockade through inhibition of the PD-1/PD-L1 pathway is a state-of-the-art cancer immunotherapy (43). In contrast, the clinical significance of PD-L2 is seldom investigated (44). The role of PD-L2 in modulating the anti-tumor immune response remains controversial (19). PD-L2 inhibits the Crohn-like lymphoid reaction and adaptive immune response during colorectal carcinogenesis (18). In addition, PD-L2 was reportedly upregulated in myeloid-derived suppressor cells with the potential to inhibit anti-tumor immunity and promote tumor growth (45). Previous analyses of The Cancer Genome Atlas (TCGA) dataset revealed that the expression of PD-L2, rather than PD-L1, was positively associated with immune-related gene expression in renal cell carcinoma and lung squamous cell carcinoma (46). Furthermore, PD-L2 was expressed independently of PD-L1 expression, providing limited value for the prediction of anti-PD-1/PD-L1 therapy responses during cancer treatment (19). Although the constitutive expression and binding affinity of PD-L2 are low (24, 47, 48), our findings support that PD-L2 may play an important role in lung carcinogenesis.

Positive correlations between *Mycobacterium tuberculosis* infection and lung cancer risk were previously reported (49, 50). TB infection can cause chronic inflammation, which may not only lead to innate and adaptive immune responses but may also be associated with immune-related gene expression (51). In this study, pulmonary TB infection was an environmental exposure associated with LUAD risk in never-smoking women. Furthermore, *PDCD1LG2* SNPs rs12237624 and rs78096119 had a significant correlation with pulmonary TB infection in relation to lung carcinogenesis. The underlying mechanisms potentially bridging the immune response to TB infection with lung carcinogenesis require further investigation, especially in the TB-endemic areas. Importantly, this finding highlights the importance of gene-environment interaction in relation to lung carcinogenesis for never-smokers.

In our study, the risk alleles of *PDCD1LG2* rs2381282, rs12237624, and rs78096119 were negatively associated with PD-L2 expression in non-tumor tissue, but not in tumor tissue. The expression and prognostic value of PD-L2 expression in lung cancer have been previously reported (52–54). The interaction between PD-L2 and PD-1 inhibits strong B7-CD28 signals at low antigen concentrations. At high antigen concentrations, the interaction between PD-L2 and PD-1 reduced cytokine production but did not inhibit T cell proliferation (55). The correlation between these *PDCD1LG2* SNPs and PD-L2 expression requires further investigation, which might provide further insight into the PD-1/PD-L2 axis in lung carcinogenesis.

The current study has several limitations. First, this multi-center study was hospital-based. The number of participants was considerably smaller than those in population-based studies. Second, no independent data validation was carried out. Since the proportion of never-smokers in most population-based studies on lung carcinogenesis has been relatively small, large studies in never-smokers are necessary to validate the current findings. Third, our healthy controls were recruited from the health examination departments of six hospitals, which may result in a healthy volunteer effect. Therefore, the current results should be interpreted cautiously.

In conclusion, we identified novel *PDCD1LG2* SNPs significantly correlated with LUAD risk in never-smoking women. Of note, some of the identified SNPs interacted with pulmonary TB infection in relation to lung carcinogenesis. These findings may help stratify a high-risk population in never-smokers for early detection of lung cancer.

## Data Availability Statement

The datasets presented in this study can be found in online repositories. The names of the repository/repositories and accession number(s) can be found in the article/**Supplementary Material**.

## Ethics Statement

The protocol of the study was approved by Research Ethics Committee, National Taiwan University Hospital, Taipei, Taiwan; Institutional Review Board, Taipei Veterans General Hospital, Taipei, Taiwan; Chang Gung Medical Foundation Institutional Review Board, Taipei, Taiwan; Institutional Review Board, Taichung Veterans General Hospital, Taichung, Taiwan; Institutional Review Board, National Cheng Kung University Hospital, Tainan, Taiwan; Institutional Review Board, Kaohsiung Medical University Chung-Ho Memorial Hospital, Kaohsiung, Taiwan; Research ethics committee, National Health Research Institutes, Zhunan, Taiwan; Institutional Review Board, China Medical University Hospital, Taichung, Taiwan. The obtained consents from all participants were written in the study. The patients/participants provided their written informed consent to participate in this study.

## Author Contributions

CH, I-SC, W-CS, K-YC, Y-HT, C-JC, and P-CY conceived the study and designed the experiments. CH, I-SC, K-YC, L-HC, S-KL, H-HH, and P-CY contributed to study design and statistical analyses. H-CL, W-TF, and S-SJ contributed to the bioinformatics analysis. CH, W-CS, G-CC, K-YC, Y-MC, M-SH, Y-HT, and P-CY contributed reagents and materials. S-KL, L-HC, and K-YC wrote the first draft of the manuscript. C-YC contributed to the collection of tumor specimens for eQTL analysis. W-CS, G-CC, K-YC, Y-MC, M-SH, Y-HT, and P-CY contributed to the recruitment of subjects. All authors contributed to the article and approved the submitted version.

## Funding

The GELAC study was granted by the National Health Research Institutes (NHRI) and by the Ministry of Sciences and Technology of Taiwan (grant no. MOST 106-2319-B-400-001).

## Conflict of Interest

S-KL received honoraria for speech from Roche, AstraZeneca, Pfizer, Merck Sharp & Dohme, Novartis, and Boehringer Ingelheim. K-YC received honoraria for speech from Pfizer, Novartis, Merck Sharp & Dohme, AstraZeneca, Roche, Boehringer Ingelheim, Eli Lilly, Chugai Pharmaceutical, and Bristol-Myers Squibb, as well as travel/accommodation/meeting expenses from Merck Sharp & Dohme, Chugai Pharmaceutical, and Boehringer Ingelheim. Y-MC served on advisory boards of Merck Sharp & Dohme, Ono Pharmaceutical, Astra-Zeneca, Roche, Boehringer Ingelheim, and Bristol-Myers Squibb.

The remaining authors declare that the research was conducted in the absence of any commercial or financial relationships that could be construed as a potential conflict of interest.

## Publisher’s Note

All claims expressed in this article are solely those of the authors and do not necessarily represent those of their affiliated organizations, or those of the publisher, the editors and the reviewers. Any product that may be evaluated in this article, or claim that may be made by its manufacturer, is not guaranteed or endorsed by the publisher.

## Abbreviations

COPD, chronic obstructive pulmonary disease; EGFR, epidermal growth factor receptor; ETS, environmental tobacco smoking; eQTL, expression quantitative trait loci; GELAC, Genetic Epidemiological Study of Lung Adenocarcinoma; GWAS, genome-wide association study; LD, linkage disequilibrium; LUAD, lung adenocarcinoma; NSCLC, non-small cell lung cancer; PD-1, programmed death-1; PD-L1, programmed death-ligand 1; PD-L2, programmed death-ligand 2; SNP, single nucleotide polymorphism; TB, tuberculosis; TCGA, The Cancer Genome Atlas.
