# Supplementary material for: Programmed Death Ligand 2 Gene Polymorphisms Are Associated With Lung Adenocarcinoma Risk in Female Never-Smokers
Source: Front Oncol. 2021 Sep 24;11:753788. doi: 10.3389/fonc.2021.753788 (PMC8497977; doi:10.3389/fonc.2021.753788)
Supplement: Supplementary file 1 [file Table_1.docx]

Supplementary Table S1. Gene range and numbers of analyzed SNPs within *PDCD1*, *CD274*, and *PDCD1LG2*

| Gene name | Gene range | Total SNP numbers | SNPs passing genotype quality control^a^ |
| --- | --- | --- | --- |
| *PDCD1* | chr2: 242,792,033–242,801,060 | 344 | 36 (5) |
| *CD274* | chr9: 5,450,503–5,470,566 | 606 | 58 (9) |
| *PDCD1LG2* | chr9: 5,510,512–5,571,282 | 1928 | 137 (21) |

Abbreviations: SNP, single nucleotide polymorphism.

^a^ Minor allele frequency > 0.01 (numbers of tagged SNPs with a PLINK pruning threshold of R^2^ < 0.2).

Supplementary Table S2. Correlation between identified *PDCD1LG2* SNPs and pulmonary TB history is associated with lung adenocarcinoma risk

| SNP | Allele^a^ | With TB | Multivariate OR^b^ | 95%CI^b^ | *p*-value for correlation^b^ |
| --- | --- | --- | --- | --- | --- |
| rs2381282^#^ | T/C | Yes | 0.553 | (0.148, 2.058) | 0.266 |
|  |  | No | 1.172 | (1.010, 1.361) |  |
| rs4742103^#^ | C/T | Yes | 1.555 | (0.503, 4.811) | 0.244 |
|  |  | No | 0.788 | (0.663, 0.937) |  |
| rs4237162 | C/T | Yes | 0.589 | (0.214, 1.626) | 0.135 |
|  |  | No | 1.292 | (1.079, 1.548) |  |
| rs4742104^#^ | C/T | Yes | 0.716 | (0.267, 1.924) | 0.766 |
|  |  | No | 0.834 | (0.716, 0.971) |  |
| rs12237624^#^ | C/T | Yes | 0.247 | (0.044, 1.398) | 0.040 |
|  |  | No | 1.579 | (1.094, 2.279) |  |
| rs78096119^#^ | A/G | Yes | 0.216 | (0.038, 1.240) | 0.031 |
|  |  | No | 1.533 | (1.084, 2.167) |  |
| rs6476988^#^ | A/G | Yes | 1.133 | (0.390, 3.291) | 0.939 |
|  |  | No | 1.182 | (1.002, 1.395) |  |
| rs7857315^#^ | T/C | Yes | 1.132 | (0.390, 3.291) | 0.924 |
|  |  | No | 1.194 | (1.012, 1.409) |  |
| rs10975178^#^ | A/G | Yes | 0.929 | (0.328, 2.631) | 0.631 |
|  |  | No | 1.202 | (1.023, 1.413) |  |
| rs7854413 | C/T | Yes | 0.461 | (0.145, 1.469) | 0.408 |
|  |  | No | 0.760 | (0.597, 0.968) |  |
| rs56001683^#^ | G/T | Yes | 0.588 | (0.182, 1.900) | 0.621 |
|  |  | No | 0.795 | (0.631, 1.002) |  |
| rs7858319^#^ | C/A | Yes | 0.588 | (0.182, 1.900) | 0.622 |
|  |  | No | 0.795 | (0.629, 1.003) |  |

Abbreviations: CI, confidence interval; MAF, minor allele frequencies; OR, odds ratio; SNP, single nucleotide polymorphism; TB, tuberculosis.

^a^ Coding allele/Reference allele

^b^ Covariates of age, education levels, body mass index, first-degree family with a history of lung cancer, history of pulmonary tuberculosis infection, cooking time in years, cooking with fume extractor, and environmental tobacco smoking exposure were used as adjusted variables.

^#^ Imputed SNP

Supplementary Table S3. Results of eQTL analyses for 12 identified *PDCD1LG2* SNPs in lung tumor and adjacent normal tissues

|  |  | Chr:Position  (GRCH37/hg19) |  | *PDCD1LG2* | | |
| --- | --- | --- | --- | --- | --- | --- |
| SNP | Minor allele |  | Tissue | beta |  | *p*-value |
| rs2381282^#^ | T | 9:5514839 | Normal | -0.148 |  | < 0.001 |
|  |  |  | Tumor | -0.059 |  | 0.232 |
| rs4742103^#^ | C | 9:5526077 | Normal | 0.050 |  | 0.260 |
|  |  |  | Tumor | -0.048 |  | 0.373 |
| rs4237162 | C | 9:5526124 | Normal | -0.053 |  | 0.259 |
|  |  |  | Tumor | -0.033 |  | 0.568 |
| rs4742104^#^ | C | 9:5532871 | Normal | -0.051 |  | 0.189 |
|  |  |  | Tumor | 0.032 |  | 0.500 |
| rs12237624^#^ | C | 9:5549080 | Normal | -0.247 |  | 0.019 |
|  |  |  | Tumor | 0.111 |  | 0.391 |
| rs78096119^#^ | A | 9:5555489 | Normal | -0.247 |  | 0.019 |
|  |  |  | Tumor | 0.111 |  | 0.391 |
| rs6476988^#^ | A | 9:5555554 | Normal | 0.057 |  | 0.194 |
|  |  |  | Tumor | 0.008 |  | 0.882 |
| rs7857315^#^ | T | 9:5555897 | Normal | 0.057 |  | 0.194 |
|  |  |  | Tumor | 0.008 |  | 0.882 |
| rs10975178^#^ | A | 9:5556434 | Normal | 0.053 |  | 0.233 |
|  |  |  | Tumor | -0.034 |  | 0.530 |
| rs7854413 | C | 9:5557708 | Normal | 0.118 |  | 0.169 |
|  |  |  | Tumor | 0.041 |  | 0.691 |
| rs56001683^#^ | G | 9:5559495 | Normal | 0.134 |  | 0.106 |
|  |  |  | Tumor | 0.070 |  | 0.487 |
| rs7858319^#^ | C | 9:5564748 | Normal | 0.134 |  | 0.106 |
|  |  |  | Tumor | 0.070 |  | 0.487 |

Abbreviations: eQTL, expression quantitative trait loci; SNP, single nucleotide polymorphism.

^#^Imputed SNP
